# TGF-β Inhibitors for Therapeutic Management of Kidney Fibrosis

**DOI:** 10.3390/ph15121485

**Published:** 2022-11-29

**Authors:** Cheol Ho Park, Tae-Hyun Yoo

**Affiliations:** Department of Internal Medicine, Institute of Kidney Disease Research, College of Medicine, Yonsei University, Seodaemun-gu, Seoul 03722, Republic of Korea

**Keywords:** TGF-β, kidney, fibrosis

## Abstract

Kidney fibrosis is a common pathophysiological mechanism of chronic kidney disease (CKD) progression caused by several underlying kidney diseases. Among various contributors to kidney fibrosis, transforming growth factor-β1 (TGF-β1) is the major factor driving fibrosis. TGF-β1 exerts its profibrotic attributes via the activation of canonical and non-canonical signaling pathways, which induce proliferation and activation of myofibroblasts and subsequent accumulation of extracellular matrix. Over the past few decades, studies have determined the TGF-β1 signaling pathway inhibitors and evaluated whether they could ameliorate the progression of CKD by hindering kidney fibrosis. However, therapeutic strategies that block TGF-β1 signaling have usually demonstrated unsatisfactory results. Herein, we discuss the therapeutic concepts of the TGF-β1 signaling pathway and its inhibitors and review the current state of the art regarding regarding TGF-β1 inhibitors in CKD management.

## 1. Introduction

Chronic kidney disease (CKD) is a global public health issue, and the medical expenses due to CKD are substantial as it advances to kidney failure with replacement therapy (KFRT) [1,2,3]. Given the disease burden of CKD, the development and implementation of an effective therapeutic strategy based on its pathophysiology are imperative.

Kidney fibrosis, the most common pathophysiologic process in CKD progression, is characterized by excessive deposition of extracellular matrix (ECM) in the tubulointerstitium and ensuing kidney function impairment resulting from functional tissue loss [4,5,6,7,8,9,10]. Numerous studies have revealed the mediators of kidney fibrosis, including transforming growth factor-β1 (TGF-β1), connective tissue growth factor, and CC motif chemokine 2; and TGF-β1 has been established as the ‘master regulator’ that plays a pivotal role in renal fibrosis and subsequent kidney function deterioration [11,12,13,14].

Treatment options directed at blocking TGF-β1 itself or TGF-β1 signaling have been dynamically investigated in the past few decades. Unfortunately, these studies failed to demonstrate satisfactory results in patients with CKD [15,16,17,18]. Furthermore, TGF-β1 inhibition may result in diverse adverse events because it is a pleiotropic cytokine that contributes to several biological processes, including development, differentiation, regulation and homeostasis of immune cells, cell proliferation, autophagy, and apoptosis [19,20,21,22,23]. However, examination and development of novel therapeutic strategies for kidney fibrosis targeting TGF-β1 are still ongoing.

In this review, we discuss the role of TGF-β1 in kidney fibrosis and emphasize the new therapeutic opportunities for inhibition of TGF-β1 or TGF-β1-associated signaling pathways for alleviating kidney fibrosis.

**Figure 1:**
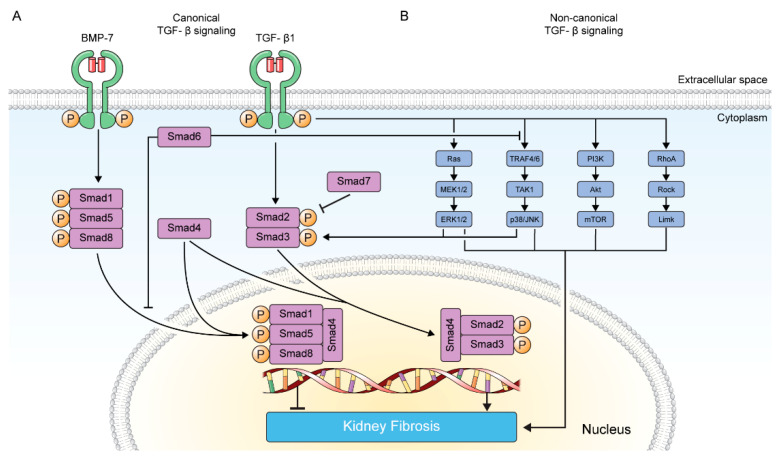
Canonical and Non-canonical TGF-β Signaling in Kidney Fibrosis. TGF-β binds to TGF-β receptors and induces signal transduction via canonical (**A**) and non-canonical (**B**) TGF-β signaling pathways. The canonical pathway includes TGF-β/Smad pathway. The non-canonical pathway includes MAP kinase, p38/JNK, PI3K/Akt, and RhoA GTPase signaling pathways.

## 2. TGF-β Signaling in Kidney Fibrosis

TGF-β, a highly conserved cytokine family in the animal kingdom and comprised of 3 isoforms (TGF-β1, TGF-β2, and TGF-β3) in mammals, plays a varied range of signaling functions, including proliferation, differentiation, and homeostasis [24,25,26]. TGF-β is synthesized in a precursor form and cleaved during the secretory process. TGF-β, in a dimeric form, mediates kidney fibrosis via canonical and non-canonical signaling pathways [14,27,28,29]. The canonical TGF-β signaling pathway refers to the TGF-β/Smad pathway, and the non-canonical TGF-β signaling pathway includes the MAP kinase, p38/ JNK, PI3K/Akt, and RhoA GTPase pathways [30].

### 2.1. Canonical TGF-β Signaling Pathway

Upon association with TGF-β, a homodimeric TGF-β type II receptor (TGFβRII) recruits and phosphorylates TGF-β type I receptor (TGFβRI), thereby activating the TGFβRI kinase property. Activated TGFβRI activates Smad2/3 by phosphorylation at C-terminal Ser-X-Ser motif, and then the phosphorylated Smad2/3 forms a complex with Smad4 [31,32,33]. This trimeric complex translocates into the nucleus and induces transcription of profibrotic molecules, including α-smooth muscle actin (α-SMA), type I collagen, and tissue inhibitor of matrix metalloproteinases (Figure 1).

BMP-7, another crucial member of the TGF-β superfamily, binds BMP receptors and subsequently phosphorylates Smad1, Smad5, and Smad8, forming heteromeric complexes with Smad4. The complex translocates into the nucleus and exerts antifibrotic effects by inhibiting Smad3-dependent gene transcription, thereby promoting transcription of antifibrotic molecules (Figure 1).

Smad6 and Smad7 are referred as inhibitory Smads. Smad6 inhibits binding of Smad4 to the BMP-7 activated Smad1/5/8 complex, thus, inhibiting BMP-7 signaling [34,35]. Additionally, Smad6 hinders TRAF6 ubiquitylation and activation, which are involved in the non-canonical TGF-β signaling pathway. Smad7 recruits Smurfs which promote TGFβRI ubiquitylation and degradation and competes with Smad2/3 for interacting with TGFβRI, thus, preventing Smad2/3 activation and propagation of the signaling [30,36,37] (Figure 1).

### 2.2. Non-Canonical TGF-β Signaling Pathway

#### 2.2.1. MAP Kinase Pathway

TGF-β-induced Ras activation leads to sequential activation of MEK1/2 and ERK1/2. Activated ERK1/2 phosphorylates Smad2 at Thr^8^ on the N-terminus and Smad3 at the linker region (Ser^204^, Ser^208^, and Thr^179^), thus, promoting and repressing TGF-β signaling, respectively [38,39]. It can also phosphorylate diverse transcription factors that contribute to kidney fibrosis and promote epithelial–mesenchymal transition and production of ECM (Figure 1).

#### 2.2.2. p38/JNK Pathway

Upon TGF-β binding, the TGF-β receptor interacts with TRAF4/6, resulting in ubiquitylation, which could be inhibited by Smad6. Ubiquitylated TRAF4/6 activates TAK1, which, in turn, activates p38/JNK. Activated p38/JNK phosphorylates Smad2/3 at their linker region (Ser^245^ and Ser^204^, respectively), subsequently enhancing TGF-β signaling [40]. Furthermore, p38/JNK phosphorylates their target transcription factors, such as c-Jun and AP-1, exhibiting a profibrotic effect (Figure 1).

#### 2.2.3. PI3K/Akt Pathway

TGF-β promotes PI3K activation through direct interaction between the TGF-β receptor and PI3K [41]. Activated PI3K phosphorylates Akt, which induces mTOR activation. This signaling pathway promotes proliferation and inhibits fibroblast apoptosis (Figure 1).

#### 2.2.4. RhoA GTPase Pathway

TGF-β induces RhoA GTPase activation, which eventually results in actin cytoskeleton remodeling via the Rock-Limk pathway and successive myofibroblast generation [42] (Figure 1).

## 3. Targeting TGF-β in Kidney Fibrosis

In this chapter, we discuss the results from preclinical studies or clinical trials using diverse TGF-β or TGF-β signaling pathway inhibitors. The key finding from clinical trials and brief summary of ongoing studies in patients are summarized in Table 1.

Of note, clinical studies evaluated the effect of TGF-β or TGF-β signaling pathway inhibitors via measuring estimated glomerular filtration rate (eGFR) and/or urine protein-to-creatinine ratio (UPCR), because those markers represent kidney function and the degree of kidney injury and may reflect the degree of kidney fibrosis [43,44,45,46].

### 3.1. Direct Inhibition of TGF-β-TGF-β Receptor Interaction

Among numerous approaches that inhibit TGF-β for moderating kidney fibrosis, direct inhibition of TGF-β and TGF-β receptor interaction is the simplest and most conventional strategy. TGF-β is a multifunctional cytokine that regulates various physiological and pathological processes. Hence, the general blockade of TGF-β may lead to de novo formation of malignant neoplasms or autoimmune diseases development [47,48,49,50,51].

#### 3.1.1. Fresolimumab

**Figure 2:**
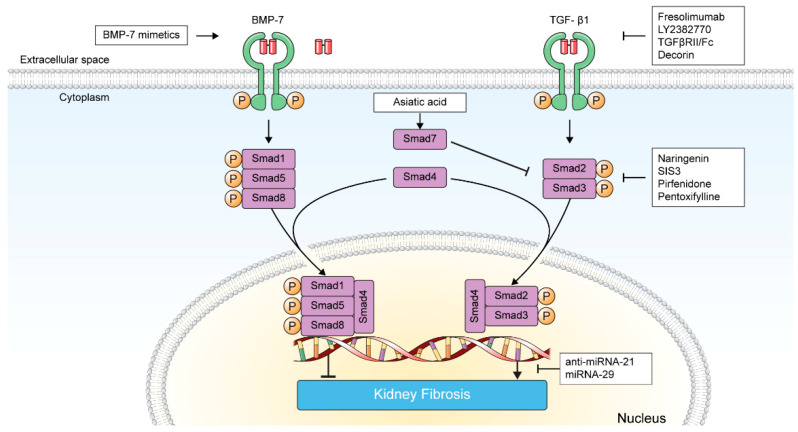
Therapeutic approaches to inhibit TGF-β/Smad-induced kidney fibrosis. Anti-TGF-β antibodies, TGFβRII/Fc, and decorin directly influence TGF-β and TGF-β receptor bindings. BMP-7 mimetics, asiatic acid, naringenin, SIS3, pirfenidone, and pentoxifylline inhibit TGF-β/Smad signaling. Anti-miRNA-21 oligonucleotides and miRNA-29 modulate the transcription product of TGF-β/Smad signaling pathway.

Fresolimumab is a humanized monoclonal antibody that neutralizes all three TGF-β isoforms (Figure 2).

Therapeutic administration of murine pan-specific TGF-β-neutralizing monoclonal antibody (1D11), a mouse analog of fresolimumab, reduces TGF-β expression and type III collagen deposition, ameliorated tubular apoptosis, and normalized tissue hypoxia in a murine model of cyclosporin nephropathy [52]. Additionally, the use of 1D11 prevented ultrastructural changes of glomerular filtration barrier in Dahl salt-sensitive rat and preserved podocyte number with reducing glomerulosclerosis in diabetic rats [53,54].

In a phase I clinical trial in patients with treatment-resistant primary focal segmental glomerulosclerosis (FSGS), a single dose of fresolimumab up to 4 mg/kg was safe and well tolerated [55]. In a phase II, double-blind, randomized clinical trial that enrolled patients with steroid-resistant primary FSGS (median eGFR 63 mL/min/1.73 m^2^; median UPCR 6.19 g/gCr), the participants received a placebo or 1 or 4 mg/kg fresolimumab for 112 days and followed up for 252 days. The primary efficacy endpoint was the proportion of patients achieving remission proteinuria (partial remission, 50% reduction in UPCR; complete remission, UPCR <300 mg/gCr). The study was terminated before registering the initially planned number of patients (planned, 88; registered, 36). None of the prespecified efficacy endpoints for proteinuria remission were attained. However, the mean percent change in the urinary protein excretion rate, assessed by UPCR, was +9.0% (*p* = 0.91), −18.5% (*p* = 0.008), and +10.5% (*p* = 0.52) in patients treated with placebo, 1 mg/kg of fresolimumab, and 4 mg/kg of fresolimumab, respectively, on day 112. Additionally, there was a non-significant but greater eGFR decline in the placebo group than in either of the fresolimumab-treated groups during the follow-up period [16].

#### 3.1.2. LY2382770

LY2382770 is a TGF-β1-specific, humanized, neutralizing monoclonal antibody (Figure 2).

In a phase II clinical trial in patients with moderate to advanced diabetic nephropathy receiving renin–angiotensin system blockade (mean eGFR 35.5 mL/min/m^2^; mean UPCR 3.3 g/gCr), the participants were scheduled to receive a placebo or 2, 10, or 50 mg of LY2382770 monthly dosing for 12 months. Although no safety issues were noted, administration of 2, 10, or 50 mg of LY2382770 failed to exhibit efficacy regarding changes in the serum creatinine level, eGFR, and UPCR. The difference in eGFR from baseline to end of treatment did not vary between placebo (−3.39 ± 5.47 mL/min/1.73 m^2^) and LY238770 treatment groups (−5.38 ± 6.27 mL/min/1.73 m^2^, −5.38 ± 7.55 mL/min/1.73 m^2^, and −4.71 ± 8.84 mL/min/1.73 m^2^, for 2, 10, and 50 mg doses, respectively). The study using LY2382770 was terminated 4 months early [15].

**Table 1:** Overview of studies for TGF-β or TGF-β signaling pathway inhibitors in patients with kidney disease.

Agent	ClinicalTrial.gov Identifier	Notes	Reference
Fresolimumab	NCT00464321	Treatment-resistant primary FSGS ^1^ N = 16 Fresolimumab was safe and well tolerated	[55]
	NCT01665391	Steroid resistant primary FSGS N = 36 Non-significant but greater eGFR ^2^ decline in the placebo group than in either of the fresolimumab-treated groups	[16]
LY2382770	NCT01113801	Diabetic nephropathy N = 258 LY2382770 did not slow progression of diabetic nephropathy	[15]
VPI-2690B	NCT02251067	Diabetic nephropathy N = 165 VPI-2690B failed to improve change in serum creatinine level	[56]
THR-184	NCT01830920	Cardiac surgery requiring CPB ^3^ N = 401 Administration of perioperative THR-184 failed to demonstrate beneficial effects on kidney function	[57]
Pirfenidone	NCT00001959	FSGS N = 18 The decline in eGFR improved after pirfenidone treatment	[17]
	NCT00063583	Diabetic nephropathy N = 77 eGFR change was not statistically different between the placebo and pirfenidone groups	[18]
	NCT04258397	CKD ^4^ (eGFR ≥ 20 mL/min/1.73 m^2^) N = 200 (Recruiting)	[58]
Pentoxifylline	NCT00285298	CKD with proteinuria (≥1 g/24 h) N = 40 Pentoxifylline group showed significantly slower eGFR decline compared with the placebo group	[59]
	-	Diabetic nephropathy N = 169 Addition of pentoxifylline to RAS ^6^ inhibitors resulted in a smaller decrease in eGFR and a greater reduction in residual albuminuria	[60]
	-	CKD (eGFR < 60 mL/min/1.73 m^2^) N = 91 Pentoxifylline decreased inflammatory markers in CKD and stabilized renal function	[61]
	NCT03625648	Diabetic nephropathy N = 2510 (recruiting) Primary outcome: Time to KFRT ^5^ or death	[62]
	NCT05487755	Diabetic nephropathy N = 90 (planned) Primary outcome: Change in serum creatinine and urine albumin-to-creatinine ratio	[63]

^1^ FSGS, focal segmental glomerulosclerosis. ^2^ eGFR, estimated glomerular filtration rate. ^3^ CBP, cardiopulmonary bypass. ^4^ CKD, chronic kidney disease. ^5^ KFRT, kidney failure with replacement therapy. ^6^ RAS, renin–angiotensin system.

#### 3.1.3. TGFβRII/Fc

TGFβRII/Fc is a chimeric protein that comprises the extracellular portion of TGFβRII and an immunoglobulin heavy-chain Fc fragment. Compared with inactive soluble TGFβRII, which has approximately 10-fold lower binding affinity for TGF-β1, TGFβRII/Fc effectively blocks the TGF-β1 binding with cell surface TGFβRII (Figure 2) [64,65].

Therefore, the administration of TGFβRII/Fc was assessed in cultured normal rat kidney cells and rat model of proliferative glomerulonephritis. TGFβRII/Fc lessened the TGF-β1-induced production of fibronectin in the rat kidney cells. Introduction of TGFβRII/Fc cDNA into the muscle of the nephritic rat using the hemagglutinating virus suppressed the glomerular TGF-β expression and resulted in ECM production in the kidney [66]. As TGFβRII/Fc binds TGF-β1 and TGF-β3 but not TGF-β2, which has antifibrotic effects, TGFβRII/Fc may have more favorable antifibrotic potency than TGF-β1 antibodies.

#### 3.1.4. Decorin

Decorin, a matrix proteoglycan induced by TGF-β, can bind to the three isoforms of TGF-β and neutralize their biological activity (Figure 2) [67]. Several studies have also depicted that decorin deficiency aggravates diabetic nephropathy in a mouse model [68,69].

Based on these attributes, a few studies have evaluated the efficacy of decorin in mouse models of kidney disease. In an experimental rat model of glomerulonephritis, administration of decorin suppressed deposition of fibronectin in glomeruli and prevented development of proteinuria [70]. Additionally, transfer of the decorin gene into skeletal muscle increased the amount of decorin in kidney, reduced TGF-β1 expression and ECM accumulation in kidney and attenuated proteinuria via ligand trapping in a rat model of glomerulonephritis [71].

#### 3.1.5. VPI-2690B

VPI-2690B is a monoclonal antibody targeting αVβ3 integrin, which is involved in TGF-β signaling [72,73]. A phase II clinical trial was conducted, aimed to evaluate safety and efficacy of VPI-2690B in diabetic nephropathy (NCT02251067) [56,74,75]. However, the study failed to demonstrate improvement in primary outcome, which was defined as a change in serum creatinine level from baseline to 12 months [76].

### 3.2. Inhibition of TGF-β Signaling

#### 3.2.1. BMP-7 and BMP-7 Agonists

BMP-7 is often referred to as an ‘intrinsic inhibitor of TGF-β’ [77,78]. BMP-7 exerts its antifibrotic effect via activation of Smad1/Smad5 and subsequent inhibition of Smad3-dependent gene transcription as described above, as well as Smad-independent pathways, such as ERK and p38 [78,79,80,81]. While BMP-7 expression levels are suppressed in various chronic kidney diseases, BMP-7 and its agonists have been actively investigated to ameliorate kidney fibrosis or facilitate kidney regeneration (Figure 2) [78,82,83,84].

**Figure 3:**
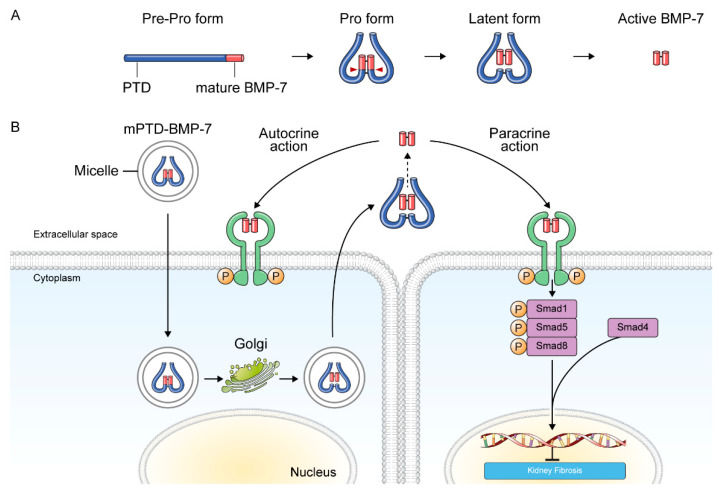
Delivery and action mechanism of mPTD-BMP-7. (**A**) Schematic diagram and preparation of PTD-BMP-7. (**B**) mPTD-BMP-7 is transduced into cells via an endosomal pathway, wherein it undergoes activation, followed by secretion. It functions in an autocrine and paracrine manner.

In cultured tubular epithelial cells, BMP-7 prevents TGF-β1 induced epithelial-mesenchymal transition by antagonizing TGF-β1-induced upregulation of α-smooth muscle actin and TGF-β1-induced downregulation of E-cadherin [78,85]. In animal models, BMP-7 re-expression is linked with spontaneous kidney regeneration [86,87].

Administration of exogenous BMP-7 or its overexpression alleviates kidney fibrosis in several CKD experimental models, including genetic models of kidney fibrosis (Alport syndrome and lupus nephritis-like glomerulonephritis), unilateral ureteral obstruction (UUO), and diabetic nephropathy [88,89,90]. Using two genetic models for chronic kidney disease and fibrosis (mice deficient in the α3-chain of type IV collagen and MRL/MpJ^lpr/lpr^ lupus mice), Zeisberg et al. showed that treatment with recombinant human BMP-7 reduced the expression of profibrotic molecules, including type I collagen and fibronectin in renal fibroblasts, and induced active matrix metalloproteinase-2 expression, which mediates removal of fibrotic matrix [88]. In a rat model of UUO, soluble BMP-7 administration decreased tubular atrophy and enhanced GFR restoration compared with vehicle or enalapril treatment [89]. In a mouse model of streptozocin-induced diabetic kidney disease, overexpression of BMP-7 through plasmid transfer activated the Smad 1/5 signaling pathway and thereby alleviated epithelial–mesenchymal transition and kidney fibrosis [90]. However, therapeutic use of BMP-7 in clinical practice is still deficient mostly owing to pharmacokinetic limitations and technical challenges, similar to other BMPs. Recombinant human BMP-7 exhibits a half-life of 7–16 min in a non-human primate model owing to enzymatic degradation and rapid clearance, demanding large amounts [91]. Furthermore, manufacturing large volumes of bioactive BMP-7 is challenging, and its efficient delivery into target tissues or organs is another obstacle [92].

Recently, a small molecule (AA123), which exerts BMP-7 mimetic activity by activating activin-like kinase 3 signaling, exhibited antifibrotic activity in murine models of nephrotoxic serum-induced chronic kidney fibrosis and diabetic nephropathy [93]. In this study, AA123 also depicted a favorable pharmacokinetic profile compared with recombinant human BMP-7. Recently, we developed a novel BMP-7 delivery system and reported the therapeutic efficacy of protein transduction domain-fused BMP-7 in micelles (mPTD- BMP-7) in UUO-induced kidney fibrosis in murine and swine models [94]. mPTD-BMP-7 was designed for endosomal transduction into cells and subsequently activated into active BMP-7 [95,96,97]. Furthermore, mPTD-BMP-7 is secreted via exosomes and exerts pharmacological effects in an autocrine and paracrine manner with a durable pharmacokinetic profile [94] (Figure 3). Herein, mPTD-BMP-7 antagonized TGF-β-mediated epithelial-mesenchymal transition via Smad1/5/8 activation and attenuated kidney fibrosis in UUO model in mice and pigs. Similarly, delivery of nanoparticle-encapsulated plasmid DNA expressing BMP-7 exhibited antifibrotic and pro-regenerative effects in a UUO mouse model [98].

Although the primary outcome was development of acute kidney injury within 7 days of cardiac surgery, a randomized, double-blind, placebo-controlled trial evaluated the safety and efficacy of THR-184, a BMP-7 mimetic peptide, in patients who received cardiac surgery requiring cardiopulmonary bypass with additional risk factors for acute kidney injury [57]. In the study, the incidence of safety-related outcomes was similar across all treatment groups. However, administration of perioperative THR-184 failed to demonstrate beneficial effects regarding kidney function, such as incidence, severity, or duration of acute kidney injury after cardiac surgery.

As the major culprit for hampering clinical use of BMP-7 mimetics is pharmacokinetic shortcomings, as stated previously, several studies have also been dedicated to enhancing BMP-7 delivery, as summarized in Table 2. Based on the results from previous studies wherein recombinant human BMP-7 administration offered long-term safety even in excessive doses and its systemic overexpression was tolerable in mice, BMP-7 could be an attractive therapeutic option for managing kidney fibrosis [88,99].

#### 3.2.2. Smad Agonists/Inhibitors

Smad7 inhibits the canonical TGF-β/Smad signaling pathway by competing with Smad2/3 for binding activated TGFβRI and subsequently downregulates TGF-β/Smad signaling [34,109]. Additionally, Smad7 induces IκBα to negatively regulate inflammation [110]. Thus, Smad7 was once considered an ideal therapeutic agent for kidney fibrosis and inflammation [111]. However, Smad7 overexpression results in podocyte apoptosis [112].

As Smad7 competes with Smad3 to bind to TGFβRI and blocks TGF-β/Smad signaling, restoring the balance between Smad3 and Smad7 has been proposed as a potential tactic for treating kidney fibrosis [111,113]. SIS3, a specific Smad3 inhibitor, exerted antifibrotic effects in mice with streptozotocin-induced diabetes through inhibiting endothelial–mesenchymal transition [114]. In a mouse model of UUO, treatment with SIS3 retarded the progression of kidney fibrosis by inhibiting α-smooth muscle actin expression, myofibroblast accumulation, and deposition of extracellular matrix, including type I collagen and fibronectin [115]. Furthermore, simultaneous administration of naringenin (Smad3 inhibitor) and asiatic acid (Smad7 agonist) ameliorated UUO-induced kidney fibrosis in mice via inhibition of α-smooth muscle actin and type I collagen expression with an additive effect [116].

#### 3.2.3. Pirfenidone

Pirfenidone is a small synthetic molecule initially developed and approved for treating idiopathic pulmonary fibrosis [117,118]. Pirfenidone exerts antifibrotic action by inhibiting Smad3 phosphorylation, profibrotic mediator synthesis, TGF-β1 expression, and subsequent proliferation and differentiation of fibroblasts (Figure 2) [119,120].

In a rat model of chronic cyclosporin nephrotoxicity, administration of pirfenidone decreased expression of TGF-β1, plasminogen activator inhibitor-1, and biglycan in the kidney. In the experiment, cyclosporin-induced decrease in creatinine clearance and histologic changes in the kidney also improved upon pirfenidone treatment [121]. In 5/6 six nephrectomy rat model of FSGS, long-term treatment of pirfenidone attenuated the accumulation of matrix protein in the glomerulus in addition to the reduction in glomerular TGF-β expression [122]. Furthermore, pirfenidone also prevented loss of glomerular filtration barrier and reduced fibrosis in animal models of kidney diseases, including vanadate-induced kidney fibrosis, doxorubicin-induced nephrotoxicity, diabetic nephropathy, and UUO [123,124,125,126].

In an open-label trial evaluating the safety and efficacy of pirfenidone in patients with FSGS (median eGFR 26 mL/min/1.73 m^2^; median 24 h urine protein 3.37 g), the subjects received 800 mg of pirfenidone thrice daily. The monthly eGFR slope (mL/min/1.73 m^2^/month) was compared between the baseline and treatment periods for each participant. The decline in eGFR improved from a median of −0.61 mL/min/1.73 m^2^/month (interquartile range (IQR), −1.31 to −0.41) during the baseline period, to −0.45 mL/min/1.73 m^2^/month (IQR, −0.78 to −0.16) during the treatment period, representing a 25% improvement (*p* < 0.01). However, some adverse events, including fatigue, dyspepsia, and photosensitivity dermatitis were reported [17].

In a randomized, placebo-controlled trial in patients with diabetic nephropathy (mean eGFR 38 mL/min/1.73 m^2^; median urine albumin-to-creatinine ratio 143 mg/gCr), the participants were administered a placebo or 1200 or 2400 mg/day of pirfenidone for 1 year. There was a significant difference in the eGFR change from baseline to the completion of study period between placebo and pirfenidone 1200 mg/day groups. The mean difference in eGFR change was +5.5 mL/min/1.73 m^2^ (95% confidence interval (CI), 1.1, 9.9; *p* = 0.026), representing a favorable outcome in the pirfenidone 1200 mg/day group. However, the mean difference in eGFR change between the placebo and pirfenidone 2400 mg/day groups was statistically insignificant (0.3, 95% CI −3.7, 4.2; *p* = 0.89). Additionally, eGFR change was not statistically different between the placebo and pirfenidone groups (*p* = 0.085) [18].

In addition to the clinical trials mentioned above, a randomized, double-blind, placebo-controlled, phase II interventional study in patients with CKD started in October 2020 to investigate the effect of pirfenidone on kidney fibrosis assessed by diffusion-weighted magnetic resonance imaging and urinary markers of tubulointerstitial fibrosis, as well as eGFR decline and the amount of urinary albumin excretion (NCT04258397) [58].

#### 3.2.4. Pentoxifylline

Pentoxifylline is a non-specific phosphodiesterase inhibitor that has been employed to manage intermittent claudication in peripheral vascular disease and alcoholic hepatitis [127,128]. Recently, the anti-inflammatory and antifibrotic attributes of pentoxifylline, which inhibits the Smad2/3/4 cascade and NF-κB, have been explored in diverse disease models including kidney fibrosis (Figure 2) [129,130,131,132,133,134,135].

Pentoxifylline inhibited proliferation, differentiation to myofibroblast, and extracellular matrix synthesis of primary renal fibroblasts which were established from human kidney biopsies [129]. Furthermore, pentoxifylline reduced the expression of profibrotic genes in cultured rat fibroblasts and mesangial cells upon angiotensin II or TGF-β1 treatment, and in rat proximal tubular cells stimulated by albumin or angiotensin II [130].

In a rat anti-Thy1 glomerulonephritis model, administration of pentoxifylline attenuated urinary protein and nephrin excretion, and inhibited phosphorylation of NF-κB and Smad2/5 [134]. In another study, treatment with pentoxifylline attenuated plasma creatinine elevation, proteinuria, glomerulosclerosis, and interstitial fibrosis in rat kidney induced by 5/6 subtotal nephrectomy [130].

In a randomized, placebo-controlled trial in patients with CKD (mean eGFR 34.1 or 29.5 mL/min/1.73 m^2^ for placebo or pentoxifylline group, respectively; median 24 h urine protein 2.5 or 1.9 g for placebo or pentoxifylline group, respectively), the subjects were administered a placebo or 400 mg pentoxifylline twice daily for 1 year. At the end of study, the pentoxifylline group depicted significantly slower eGFR decline (−1.2 ± 7.0 mL/min/1.73 m^2^/ year) compared with the placebo group (−7.2 ± 8.2 mL/min/1.73 m^2^/year) (*p* = 0.03) [59].

In an open-label, prospective, randomized trial to evaluate the renoprotective effect of pentoxifylline on top of renin–angiotensin system inhibitors in patients with diabetic nephropathy (mean eGFR 37.4 mL/min/1.73 m^2^; median 24 h urine albumin 1100 mg), the patients were administered 1200 mg/day of pentoxifylline for 2 years. At the end of the trial, eGFR had decreased by 6.5 ± 0.4 mL/min/1.73 m^2^ in the control, and, 2.1 ± 0.4 mL/min/1.73 m^2^ in the pentoxifylline group (between-group difference; 4.3 mL/min/1.73 m^2^, 95% CI, 3.1, 5.5; *p* < 0.001). Additionally, the daily urinary albumin excretion change was 5.7% in the control and −14.9% in the pentoxifylline group (*p* = 0.001) [60].

In a 12-month trial evaluating the effects of pentoxifylline on inflammatory parameters in patients with CKD (mean eGFR 40.1 or 42.3 mL/min/1.73 m^2^ for placebo or pentoxifylline group, respectively; median 24 h urine albumin 115 or 56 mg for placebo or pentoxifylline group, respectively), the participants received a placebo or 400 mg of pentoxifylline twice daily. The control group presented worsening of kidney function (from 40.1 ± 12.4 to 35.7 ± 13.4 mL/min/1.73 m^2^), whereas the pentoxifylline group demonstrated no significant decline in eGFR post 12 months (from 42.3 ± 10.2 to 44.7 ± 11.3 mL/min/1.73 m^2^) (*p* < 0.001, between groups) [61]. Furthermore, in a post hoc analysis of the 12-month trial following an additional 7 years, the pentoxifylline group exhibited a favorable renal outcome, defined as a doubling of serum creatinine level and/or ≥50% decrease in eGFR and/or the initiation of kidney replacement therapy [136].

In November 2019, the recruitment of more than 2000 patients with diabetic kidney disease was initiated to establish whether pentoxifylline can prevent CKD progression and reduce mortality in patients with diabetic kidney disease (NCT03625648) [62]. More recently, a randomized trial started to evaluate the safety and efficacy of pentoxifylline in diabetic nephropathy, comparing with tadalafil, a selective phosphodiesterase type 5 inhibitor, on kidney function and albuminuria (NCT05487755) [63].

### 3.3. Inhibition of TGF-β-Induced Transcription Product

#### MicroRNAs

MicroRNAs (miRNAs) are short, non-coding, single-stranded RNA molecules of approximately 20–22 nucleotides that are not transcribed into peptides [137]. miRNAs silence gene expression, the translational repression and/or targeted mRNAs degradation [138]. miRNAs play diverse roles in various biological processes, including tissue fibrosis (Figure 2) [139,140,141,142].

Several miRNA species induced by TGF-β1 exhibiting profibrotic effects have been established, the best characterized of which is miRNA-21 [14,143]. miRNA-21 is upregulated in CKD, and the severity of fibrosis or kidney function correlates with miRNA-21 expression levels in patients with diabetic nephropathy [144,145]. miRNA-21 exerts its profibrotic effect by downregulating antifibrotic genes, including Smad7, Pparα, Spry1, and Pten [143,146,147]. miRNA-21-deficient mice depicted far less interstitial fibrosis in response to UUO or unilateral ischemia–reperfusion injury than wild-type mice [148]. Anti-miRNA-21 oligonucleotide administration inhibits kidney fibrosis in UUO and *db*/*db* kidney diseases [148,149,150].

miRNA-29 is an endogenous tissue fibrosis inhibitor, which downregulates mRNA levels of diverse profibrotic molecules, including collagens, matrix metalloproteinases, and Fos. Expression level of miRNA-29 is suppressed in UUO-induced kidney fibrosis and adenine-induced CKD [151,152]. Overexpression of miRNA-29 ameliorated kidney fibrosis in a UUO mouse model [151].

Although there are challenges in the therapeutic implementation of miRNA targeting, including non-specificity, off-target effects, and toxicity, miRNA-21 downregulation or miRNA-29 overexpression appears to be a promising antifibrotic approach.

## 4. Conclusions

As outlined in this review, TGF-β plays an imperative role in kidney fibrosis via canonical and non-canonical signaling pathways, and several TGF-β inhibitors have been extensively investigated. Although there is still a wide gap between promising preclinical findings and clinical implications of efficient therapeutic agents for kidney fibrosis inhibition, we believe that effective antifibrotic agents to alleviate or even reverse CKD progression will be applicable in the foreseeable future.

## Figures and Tables

**Table 2:** Overview of studies for enhancing BMP-7 delivery employing various carriers.

Carrier Type	Preclinical Model	Reference
Collagen	Bone defects in non-human primates	[100]
	Vertebral interbody fusion in sheep	[101]
Hydroxyapatite	Orthotopic skull defects in baboons	[102]
	Spinal fusion in sheep	[103]
Poly(D,L-lactide-co-glycolide)	Bone formation from rabbit muscle cells	[104]
	Osteochondral defect in rabbit knee	[105]
CMC ^1^-Collagen	Tibial bone defects in sheep	[106]
1,4 Butane-diisocyanate-hydrogel	Femoral intramedullary injection in mice	[107]
Micelle	Unilateral ureteral obstruction in mice/pig	[94]
Chitosan nanoparticle	Unilateral ureteral obstruction in mice	[98]
	Femoral bone defect in rat	[108]

^1^ CMC, carboxymethyl cellulose.

## Data Availability

No new data were created or analyzed in this study.

## References

[1] Xie Y., Bowe B., Mokdad A.H., Xian H., Yan Y., Li T., Maddukuri G., Tsai C.Y., Floyd T., Al-Aly Z. (2018). Analysis of the global burden of disease study highlights the global, regional, and national trends of chronic kidney disease epidemiology from 1990 to 2016. Kidney Int..

[2] United States Renal Data System (2021). 2021 USRDS Annual Data Report: Epidemiology of Kidney Disease in the United States.

[3] Boenink R., Astley M.E., Huijben J.A., Stel V.S., Kerschbaum J., Ots-Rosenberg M., Asberg A.A., Lopot F., Golan E., Castro de la Nuez P. (2022). The era registry annual report 2019: Summary and age comparisons. Clin. Kidney J..

[4] Risdon R.A., Sloper J.C., De Wardener H.E. (1968). Relationship between renal function and histological changes found in renal-biopsy specimens from patients with persistent glomerular nephritis. Lancet.

[5] Bohle A., Grund K.E., Mackensen S., Tolon M. (1977). Correlations between renal interstitium and level of serum creatinine. Morphometric investigations of biopsies in perimembranous glomerulonephritis. Virchows Arch. A Pathol. Anat. Histol..

[6] Eddy A.A., Neilson E.G. (2006). Chronic kidney disease progression. J. Am. Soc. Nephrol..

[7] Mutsaers H.A.M., Norregaard R. (2022). Prostaglandin E2 receptors as therapeutic targets in renal fibrosis. Kidney Res. Clin. Pract..

[8] Bhatia D., Capili A., Choi M.E. (2020). Mitochondrial dysfunction in kidney injury, inflammation, and disease: Potential therapeutic approaches. Kidney Res. Clin. Pract..

[9] Liu Y. (2011). Cellular and molecular mechanisms of renal fibrosis. Nat. Rev. Nephrol..

[10] Zeisberg M., Kalluri R. (2013). Cellular mechanisms of tissue fibrosis. 1. Common and organ-specific mechanisms associated with tissue fibrosis. Am. J. Physiol. Cell Physiol..

[11] Sato M., Muragaki Y., Saika S., Roberts A.B., Ooshima A. (2003). Targeted disruption of TGF-beta1/Smad3 signaling protects against renal tubulointerstitial fibrosis induced by unilateral ureteral obstruction. J. Clin. Investig..

[12] Russo L.M., del Re E., Brown D., Lin H.Y. (2007). Evidence for a role of transforming growth factor (TGF)-beta1 in the induction of postglomerular albuminuria in diabetic nephropathy: Amelioration by soluble TGF-beta type II receptor. Diabetes.

[13] Wynn T.A., Ramalingam T.R. (2012). Mechanisms of fibrosis: Therapeutic translation for fibrotic disease. Nat. Med..

[14] Meng X.M., Nikolic-Paterson D.J., Lan H.Y. (2016). TGF-β: The master regulator of fibrosis. Nat. Rev. Nephrol..

[15] Voelker J., Berg P.H., Sheetz M., Duffin K., Shen T., Moser B., Greene T., Blumenthal S.S., Rychlik I., Yagil Y. (2017). Anti-TGF-beta1 antibody therapy in patients with diabetic nephropathy. J. Am. Soc. Nephrol..

[16] Vincenti F., Fervenza F.C., Campbell K.N., Diaz M., Gesualdo L., Nelson P., Praga M., Radhakrishnan J., Sellin L., Singh A. (2017). A phase 2, double-blind, placebo-controlled, randomized study of fresolimumab in patients with steroid-resistant primary focal segmental glomerulosclerosis. Kidney Int. Rep..

[17] Cho M.E., Smith D.C., Branton M.H., Penzak S.R., Kopp J.B. (2007). Pirfenidone slows renal function decline in patients with focal segmental glomerulosclerosis. Clin. J. Am. Soc. Nephrol..

[18] Sharma K., Ix J.H., Mathew A.V., Cho M., Pflueger A., Dunn S.R., Francos B., Sharma S., Falkner B., McGowan T.A. (2011). Pirfenidone for diabetic nephropathy. J. Am. Soc. Nephrol..

[19] Li M.O., Sanjabi S., Flavell R.A. (2006). Transforming growth factor-beta controls development, homeostasis, and tolerance of T cells by regulatory T cell-dependent and -independent mechanisms. Immunity.

[20] Li M.O., Wan Y.Y., Sanjabi S., Robertson A.K., Flavell R.A. (2006). Transforming growth factor-beta regulation of immune responses. Annu. Rev. Immunol..

[21] Bottinger E.P., Bitzer M. (2002). TGF-beta signaling in renal disease. J. Am. Soc. Nephrol..

[22] Meng X.M., Chung A.C., Lan H.Y. (2013). Role of the TGF-beta/BMP-7/Smad pathways in renal diseases. Clin. Sci..

[23] Basile D.P., Ullah M.M., Collet J.A., Mehrotra P. (2021). T helper 17 cells in the pathophysiology of acute and chronic kidney disease. Kidney Res. Clin. Pract..

[24] Huminiecki L., Goldovsky L., Freilich S., Moustakas A., Ouzounis C., Heldin C.H. (2009). Emergence, development and diversification of the TGF-beta signalling pathway within the animal kingdom. BMC Evol. Biol..

[25] Wrana J.L. (2013). Signaling by the TGF-beta superfamily. Cold Spring Harb. Perspect. Biol..

[26] Hinck A.P. (2012). Structural studies of the tgf-betas and their receptors—Insights into evolution of the TGF-beta superfamily. FEBS Lett..

[27] Lee M.K., Pardoux C., Hall M.C., Lee P.S., Warburton D., Qing J., Smith S.M., Derynck R. (2007). TGF-beta activates erk map kinase signalling through direct phosphorylation of shca. EMBO J..

[28] Liu M., Ning X., Li R., Yang Z., Yang X., Sun S., Qian Q. (2017). Signalling pathways involved in hypoxia-induced renal fibrosis. J. Cell. Mol. Med..

[29] ten Dijke P., Arthur H.M. (2007). Extracellular control of TGF-beta signalling in vascular development and disease. Nat. Rev. Mol. Cell Biol..

[30] Tzavlaki K., Moustakas A. (2020). TGF-β signaling. Biomolecules.

[31] Abdollah S., Macias-Silva M., Tsukazaki T., Hayashi H., Attisano L., Wrana J.L. (1997). TbetaRI phosphorylation of Smad2 on Ser465 and Ser467 is required for Smad2-Smad4 complex formation and signaling. J. Biol. Chem..

[32] Souchelnytskyi S., Tamaki K., Engstrom U., Wernstedt C., ten Dijke P., Heldin C.H. (1997). Phosphorylation of Ser465 and Ser467 in the C terminus of Smad2 mediates interaction with Smad4 and is required for transforming growth factor-beta signaling. J. Biol. Chem..

[33] Liu X., Sun Y., Constantinescu S.N., Karam E., Weinberg R.A., Lodish H.F. (1997). Transforming growth factor beta-induced phosphorylation of Smad3 is required for growth inhibition and transcriptional induction in epithelial cells. Proc. Natl. Acad. Sci. USA.

[34] Shi Y., Massague J. (2003). Mechanisms of TGF-beta signaling from cell membrane to the nucleus. Cell.

[35] Hata A., Lagna G., Massague J., Hemmati-Brivanlou A. (1998). Smad6 inhibits BMP/Smad1 signaling by specifically competing with the Smad4 tumor suppressor. Genes Dev..

[36] Kavsak P., Rasmussen R.K., Causing C.G., Bonni S., Zhu H., Thomsen G.H., Wrana J.L. (2000). Smad7 binds to Smurf2 to form an E3 ubiquitin ligase that targets the TGF beta receptor for degradation. Mol. Cell.

[37] Ebisawa T., Fukuchi M., Murakami G., Chiba T., Tanaka K., Imamura T., Miyazono K. (2001). Smurf1 interacts with transforming growth factor-beta type I receptor through Smad7 and induces receptor degradation. J. Biol. Chem..

[38] Funaba M., Zimmerman C.M., Mathews L.S. (2002). Modulation of Smad2-mediated signaling by extracellular signal-regulated kinase. J. Biol. Chem..

[39] Kretzschmar M., Doody J., Timokhina I., Massague J. (1999). A mechanism of repression of TGF-beta/Smad signaling by oncogenic ras. Genes Dev..

[40] Seong H.A., Jung H., Ha H. (2010). Murine protein serine/threonine kinase 38 stimulates TGF-beta signaling in a kinase-dependent manner via direct phosphorylation of smad proteins. J. Biol. Chem..

[41] Yi J.Y., Shin I., Arteaga C.L. (2005). Type I transforming growth factor beta receptor binds to and activates phosphatidylinositol 3-kinase. J. Biol. Chem..

[42] Bhowmick N.A., Ghiassi M., Bakin A., Aakre M., Lundquist C.A., Engel M.E., Arteaga C.L., Moses H.L. (2001). Transforming growth factor-beta1 mediates epithelial to mesenchymal transdifferentiation through a RhoA-dependent mechanism. Mol. Biol. Cell.

[43] National Kidney Foundation (2002). K/DOQI clinical practice guidelines for chronic kidney disease: Evaluation, classification, and stratification. Am. J. Kidney Dis..

[44] Burton C., Harris K.P. (1996). The role of proteinuria in the progression of chronic renal failure. Am. J. Kidney Dis..

[45] Ruggenenti P., Perna A., Mosconi L., Pisoni R., Remuzzi G. (1998). Urinary protein excretion rate is the best independent predictor of ESRF in non-diabetic proteinuric chronic nephropathies. “Gruppo Italiano di Studi Epidemiologici in Nefrologia” (gisen). Kidney Int..

[46] Eddy A.A. (2004). Proteinuria and interstitial injury. Nephrol. Dial. Transplant..

[47] Akhurst R.J. (2002). TGF-beta antagonists: Why suppress a tumor suppressor?. J. Clin. Investig..

[48] Garber K. (2009). Companies waver in efforts to target transforming growth factor beta in cancer. J. Natl. Cancer Inst..

[49] Kulkarni A.B., Huh C.G., Becker D., Geiser A., Lyght M., Flanders K.C., Roberts A.B., Sporn M.B., Ward J.M., Karlsson S. (1993). Transforming growth factor beta 1 null mutation in mice causes excessive inflammatory response and early death. Proc. Natl. Acad. Sci. USA.

[50] Yaswen L., Kulkarni A.B., Fredrickson T., Mittleman B., Schiffman R., Payne S., Longenecker G., Mozes E., Karlsson S. (1996). Autoimmune manifestations in the transforming growth factor-beta 1 knockout mouse. Blood.

[51] Li M.O., Flavell R.A. (2008). TGF-beta: A master of all T cell trades. Cell.

[52] Ling H., Li X., Jha S., Wang W., Karetskaya L., Pratt B., Ledbetter S. (2003). Therapeutic role of TGF-beta-neutralizing antibody in mouse cyclosporin a nephropathy: Morphologic improvement associated with functional preservation. J. Am. Soc. Nephrol..

[53] Dahly-Vernon A.J., Sharma M., McCarthy E.T., Savin V.J., Ledbetter S.R., Roman R.J. (2005). Transforming growth factor-beta, 20-hete interaction, and glomerular injury in dahl salt-sensitive rats. Hypertension.

[54] Benigni A., Zoja C., Campana M., Corna D., Sangalli F., Rottoli D., Gagliardini E., Conti S., Ledbetter S., Remuzzi G. (2006). Beneficial effect of TGF-beta antagonism in treating diabetic nephropathy depends on when treatment is started. Nephron. Exp Nephrol..

[55] Trachtman H., Fervenza F.C., Gipson D.S., Heering P., Jayne D.R., Peters H., Rota S., Remuzzi G., Rump L.C., Sellin L.K. (2011). A phase 1, single-dose study of fresolimumab, an anti-TGF-beta antibody, in treatment-resistant primary focal segmental glomerulosclerosis. Kidney Int..

[56] (2014). US National Library of Medicine. https://clinicaltrials.gov/ct2/show/NCT02251067.

[57] Himmelfarb J., Chertow G.M., McCullough P.A., Mesana T., Shaw A.D., Sundt T.M., Brown C., Cortville D., Dagenais F., de Varennes B. (2018). Perioperative thr-184 and aki after cardiac surgery. J. Am. Soc. Nephrol..

[58] (2020). US National Library of Medicine. https://clinicaltrials.gov/ct2/show/NCT04258397.

[59] Perkins R.M., Aboudara M.C., Uy A.L., Olson S.W., Cushner H.M., Yuan C.M. (2009). Effect of pentoxifylline on GFR decline in ckd: A pilot, double-blind, randomized, placebo-controlled trial. Am. J. Kidney Dis..

[60] Navarro-Gonzalez J.F., Mora-Fernandez C., Muros de Fuentes M., Chahin J., Mendez M.L., Gallego E., Macia M., del Castillo N., Rivero A., Getino M.A. (2015). Effect of pentoxifylline on renal function and urinary albumin excretion in patients with diabetic kidney disease: The predian trial. J. Am. Soc. Nephrol..

[61] Goicoechea M., Garcia de Vinuesa S., Quiroga B., Verdalles U., Barraca D., Yuste C., Panizo N., Verde E., Munoz M.A., Luno J. (2012). Effects of pentoxifylline on inflammatory parameters in chronic kidney disease patients: A randomized trial. J. Nephrol..

[62] Leehey D.J., Carlson K., Reda D.J., Craig I., Clise C., Conner T.A., Agarwal R., Kaufman J.S., Anderson R.J., Lammie D. (2021). Pentoxifylline in diabetic kidney disease (VA PTXRx): Protocol for a pragmatic randomised controlled trial. BMJ Open.

[63] (2022). US National Library of Medicine. https://clinicaltrials.gov/ct2/show/NCT05487755.

[64] Derynck R., Zhang Y.E. (2003). Smad-dependent and Smad-independent pathways in TGF-beta family signalling. Nature.

[65] Lin H.Y., Moustakas A., Knaus P., Wells R.G., Henis Y.I., Lodish H.F. (1995). The soluble exoplasmic domain of the type II transforming growth factor (TGF)-beta receptor. A heterogeneously glycosylated protein with high affinity and selectivity for TGF-beta ligands. J. Biol. Chem..

[66] Isaka Y., Akagi Y., Ando Y., Tsujie M., Sudo T., Ohno N., Border W.A., Noble N.A., Kaneda Y., Hori M. (1999). Gene therapy by transforming growth factor-beta receptor-IgG Fc chimera suppressed extracellular matrix accumulation in experimental glomerulonephritis. Kidney Int..

[67] Yamaguchi Y., Mann D.M., Ruoslahti E. (1990). Negative regulation of transforming growth factor-beta by the proteoglycan decorin. Nature.

[68] Williams K.J., Qiu G., Usui H.K., Dunn S.R., McCue P., Bottinger E., Iozzo R.V., Sharma K. (2007). Decorin deficiency enhances progressive nephropathy in diabetic mice. Am. J. Pathol..

[69] Merline R., Lazaroski S., Babelova A., Tsalastra-Greul W., Pfeilschifter J., Schluter K.D., Gunther A., Iozzo R.V., Schaefer R.M., Schaefer L. (2009). Decorin deficiency in diabetic mice: Aggravation of nephropathy due to overexpression of profibrotic factors, enhanced apoptosis and mononuclear cell infiltration. J. Physiol. Pharmacol..

[70] Border W.A., Noble N.A., Yamamoto T., Harper J.R., Yamaguchi Y., Pierschbacher M.D., Ruoslahti E. (1992). Natural inhibitor of transforming growth factor-beta protects against scarring in experimental kidney disease. Nature.

[71] Isaka Y., Brees D.K., Ikegaya K., Kaneda Y., Imai E., Noble N.A., Border W.A. (1996). Gene therapy by skeletal muscle expression of decorin prevents fibrotic disease in rat kidney. Nat. Med..

[72] Worthington J.J., Klementowicz J.E., Travis M.A. (2011). TGF-beta: A sleeping giant awoken by integrins. Trends Biochem. Sci..

[73] Sarrazy V., Koehler A., Chow M.L., Zimina E., Li C.X., Kato H., Caldarone C.A., Hinz B. (2014). Integrins αvβ5 and αvβ3 promote latent TGF-beta1 activation by human cardiac fibroblast contraction. Cardiovasc. Res..

[74] Breyer M.D., Susztak K. (2016). The next generation of therapeutics for chronic kidney disease. Nat. Rev. Drug Discov..

[75] Yuan Q., Tang B., Zhang C. (2022). Signaling pathways of chronic kidney diseases, implications for therapeutics. Signal Transduct. Target. Ther..

[76] Bon H., Hales P., Lumb S., Holdsworth G., Johnson T., Qureshi O., Twomey B.M. (2019). Spontaneous extracellular matrix accumulation in a human in vitro model of renal fibrosis is mediated by alphav integrins. Nephron.

[77] Li R.X., Yiu W.H., Tang S.C. (2015). Role of bone morphogenetic protein-7 in renal fibrosis. Front. Physiol..

[78] Zeisberg M., Hanai J., Sugimoto H., Mammoto T., Charytan D., Strutz F., Kalluri R. (2003). BMP-7 counteracts TGF-beta1-induced epithelial-to-mesenchymal transition and reverses chronic renal injury. Nat. Med..

[79] Wang S.N., Lapage J., Hirschberg R. (2001). Loss of tubular bone morphogenetic protein-7 in diabetic nephropathy. J. Am. Soc. Nephrol..

[80] Motazed R., Colville-Nash P., Kwan J.T., Dockrell M.E. (2008). BMP-7 and proximal tubule epithelial cells: Activation of multiple signaling pathways reveals a novel anti-fibrotic mechanism. Pharm. Res..

[81] Wang S., Hirschberg R. (2004). Bone morphogenetic protein-7 signals opposing transforming growth factor beta in mesangial cells. J. Biol. Chem..

[82] Vukicevic S., Basic V., Rogic D., Basic N., Shih M.S., Shepard A., Jin D., Dattatreyamurty B., Jones W., Dorai H. (1998). Osteogenic protein-1 (bone morphogenetic protein-7) reduces severity of injury after ischemic acute renal failure in rat. J. Clin. Investig..

[83] Zeisberg M., Shah A.A., Kalluri R. (2005). Bone morphogenic protein-7 induces mesenchymal to epithelial transition in adult renal fibroblasts and facilitates regeneration of injured kidney. J. Biol. Chem..

[84] Zeisberg M., Yang C., Martino M., Duncan M.B., Rieder F., Tanjore H., Kalluri R. (2007). Fibroblasts derive from hepatocytes in liver fibrosis via epithelial to mesenchymal transition. J. Biol. Chem..

[85] Veerasamy M., Nguyen T.Q., Motazed R., Pearson A.L., Goldschmeding R., Dockrell M.E. (2009). Differential regulation of E-cadherin and alpha-smooth muscle actin by BMP 7 in human renal proximal tubule epithelial cells and its implication in renal fibrosis. Am. J. Physiol. Ren. Physiol..

[86] Mitu G., Hirschberg R. (2008). Bone morphogenetic protein-7 (BMP7) in chronic kidney disease. Front. Biosci..

[87] Li T., Surendran K., Zawaideh M.A., Mathew S., Hruska K.A. (2004). Bone morphogenetic protein 7: A novel treatment for chronic renal and bone disease. Curr. Opin. Nephrol. Hypertens..

[88] Zeisberg M., Bottiglio C., Kumar N., Maeshima Y., Strutz F., Muller G.A., Kalluri R. (2003). Bone morphogenic protein-7 inhibits progression of chronic renal fibrosis associated with two genetic mouse models. Am. J. Physiol. Ren. Physiol..

[89] Morrissey J., Hruska K., Guo G., Wang S., Chen Q., Klahr S. (2002). Bone morphogenetic protein-7 improves renal fibrosis and accelerates the return of renal function. J. Am. Soc. Nephrol..

[90] Peng W., Zhou X., Xu T., Mao Y., Zhang X., Liu H., Liang L., Liu L., Liu L., Xiao Y. (2022). BMP-7 ameliorates partial epithelial-mesenchymal transition by restoring snon protein level via Smad1/5 pathway in diabetic kidney disease. Cell Death Dis..

[91] Lo K.W., Ulery B.D., Ashe K.M., Laurencin C.T. (2012). Studies of bone morphogenetic protein-based surgical repair. Adv. Drug Deliv. Rev..

[92] Swencki-Underwood B., Mills J.K., Vennarini J., Boakye K., Luo J., Pomerantz S., Cunningham M.R., Farrell F.X., Naso M.F., Amegadzie B. (2008). Expression and characterization of a human BMP-7 variant with improved biochemical properties. Protein Expr. Purif..

[93] Sugimoto H., LeBleu V.S., Bosukonda D., Keck P., Taduri G., Bechtel W., Okada H., Carlson W., Bey P., Rusckowski M. (2012). Activin-like kinase 3 is important for kidney regeneration and reversal of fibrosis. Nat. Med..

[94] Kim S., Jeong C.H., Song S.H., Um J.E., Kim H.S., Yun J.S., Han D., Cho E.S., Nam B.Y., Yook J.I. (2020). Micellized protein transduction domain-bone morphogenetic protein-7 efficiently blocks renal fibrosis via inhibition of transforming growth factor-beta-mediated epithelial-mesenchymal transition. Front. Pharmacol..

[95] Kim N.H., Cha Y.H., Kim H.S., Lee S.E., Huh J.K., Kim J.K., Kim J.M., Ryu J.K., Kim H.J., Lee Y. (2014). A platform technique for growth factor delivery with novel mode of action. Biomaterials.

[96] Vendeville A., Rayne F., Bonhoure A., Bettache N., Montcourrier P., Beaumelle B. (2004). HIV-1 Tat enters T cells using coated pits before translocating from acidified endosomes and eliciting biological responses. Mol. Biol. Cell.

[97] Wadia J.S., Stan R.V., Dowdy S.F. (2004). Transducible TAT-HA fusogenic peptide enhances escape of TAT-fusion proteins after lipid raft macropinocytosis. Nat. Med..

[98] Midgley A.C., Wei Y., Zhu D., Gao F., Yan H., Khalique A., Luo W., Jiang H., Liu X., Guo J. (2020). Multifunctional natural polymer nanoparticles as antifibrotic gene carriers for CKD therapy. J. Am. Soc. Nephrol..

[99] Wang S., Hirschberg R. (2003). BMP7 antagonizes TGF-beta -dependent fibrogenesis in mesangial cells. Am. J. Physiol. Ren. Physiol..

[100] Cook S.D., Wolfe M.W., Salkeld S.L., Rueger D.C. (1995). Effect of recombinant human osteogenic protein-1 on healing of segmental defects in non-human primates. J. Bone Jt. Surg. Am..

[101] Magin M.N., Delling G. (2001). Improved lumbar vertebral interbody fusion using rhOP-1: A comparison of autogenous bone graft, bovine hydroxylapatite (Bio-Sss), and BMP-7 (rhOP-1) in sheep. Spine.

[102] Ripamonti U., Crooks J., Rueger D.C. (2001). Induction of bone formation by recombinant human osteogenic protein-1 and sintered porous hydroxyapatite in adult primates. Plast. Reconstr. Surg..

[103] Blattert T.R., Delling G., Dalal P.S., Toth C.A., Balling H., Weckbach A. (2002). Successful transpedicular lumbar interbody fusion by means of a composite of osteogenic protein-1 (rhBMP-7) and hydroxyapatite carrier: A comparison with autograft and hydroxyapatite in the sheep spine. Spine.

[104] Lu H.H., Kofron M.D., El-Amin S.F., Attawia M.A., Laurencin C.T. (2003). In vitro bone formation using muscle-derived cells: A new paradigm for bone tissue engineering using polymer-bone morphogenetic protein matrices. Biochem. Biophys. Res. Commun..

[105] Kim H.J., Han M.A., Shin J.Y., Jeon J.H., Lee S.J., Yoon M.Y., Kim H.J., Choi E.J., Do S.H., Yang V.C. (2019). Intra-articular delivery of synovium-resident mesenchymal stem cells via BMP-7-loaded fibrous PLGA scaffolds for cartilage repair. J. Control. Release.

[106] Pluhar G.E., Turner A.S., Pierce A.R., Toth C.A., Wheeler D.L. (2006). A comparison of two biomaterial carriers for osteogenic protein-1 (BMP-7) in an ovine critical defect model. J Bone Jt. Surg. Br..

[107] Neuerburg C., Mittlmeier L.M., Keppler A.M., Westphal I., Glass A., Saller M.M., Herlyn P.K.E., Richter H., Bocker W., Schieker M. (2019). Growth factor-mediated augmentation of long bones: Evaluation of a BMP-7 loaded thermoresponsive hydrogel in a murine femoral intramedullary injection model. J. Orthop. Surg. Res..

[108] Mantripragada V.P., Jayasuriya A.C. (2016). Bone regeneration using injectable BMP-7 loaded chitosan microparticles in rat femoral defect. Mater. Sci. Eng. C Mater. Biol. Appl..

[109] Yan X., Chen Y.G. (2011). Smad7: Not only a regulator, but also a cross-talk mediator of TGF-beta signalling. Biochem. J..

[110] Wang W., Huang X.R., Li A.G., Liu F., Li J.H., Truong L.D., Wang X.J., Lan H.Y. (2005). Signaling mechanism of TGF-beta1 in prevention of renal inflammation: Role of Smad7. J. Am. Soc. Nephrol..

[111] Lan H.Y. (2008). Smad7 as a therapeutic agent for chronic kidney diseases. Front. Biosci..

[112] Schiffer M., Bitzer M., Roberts I.S., Kopp J.B., ten Dijke P., Mundel P., Bottinger E.P. (2001). Apoptosis in podocytes induced by TGF-beta and Smad7. J. Clin. Investig..

[113] Lan H.Y. (2012). Smads as therapeutic targets for chronic kidney disease. Kidney Res. Clin. Pract..

[114] Li J., Qu X., Yao J., Caruana G., Ricardo S.D., Yamamoto Y., Yamamoto H., Bertram J.F. (2010). Blockade of endothelial-mesenchymal transition by a Smad3 inhibitor delays the early development of streptozotocin-induced diabetic nephropathy. Diabetes.

[115] Zhang Y., Meng X.M., Huang X.R., Lan H.Y. (2018). The preventive and therapeutic implication for renal fibrosis by targetting TGF-beta/smad3 signaling. Clin. Sci..

[116] Meng X.M., Zhang Y., Huang X.R., Ren G.L., Li J., Lan H.Y. (2015). Treatment of renal fibrosis by rebalancing TGF-beta/Smad signaling with the combination of asiatic acid and naringenin. Oncotarget.

[117] Taniguchi H., Ebina M., Kondoh Y., Ogura T., Azuma A., Suga M., Taguchi Y., Takahashi H., Nakata K., Sato A. (2010). Pirfenidone in idiopathic pulmonary fibrosis. Eur. Respir. J..

[118] Mora A.L., Rojas M., Pardo A., Selman M. (2017). Emerging therapies for idiopathic pulmonary fibrosis, a progressive age-related disease. Nat. Rev. Drug Discov..

[119] Tampe D., Zeisberg M. (2014). Potential approaches to reverse or repair renal fibrosis. Nat. Rev. Nephrol..

[120] Ruwanpura S.M., Thomas B.J., Bardin P.G. (2020). Pirfenidone: Molecular mechanisms and potential clinical applications in lung disease. Am. J. Respir. Cell Mol. Biol..

[121] Shihab F.S., Bennett W.M., Yi H., Andoh T.F. (2002). Pirfenidone treatment decreases transforming growth factor-beta1 and matrix proteins and ameliorates fibrosis in chronic cyclosporine nephrotoxicity. Am. J. Transplant..

[122] Shimizu T., Fukagawa M., Kuroda T., Hata S., Iwasaki Y., Nemoto M., Shirai K., Yamauchi S., Margolin S.B., Shimizu F. (1997). Pirfenidone prevents collagen accumulation in the remnant kidney in rats with partial nephrectomy. Kidney Int. Suppl..

[123] Al-Bayati M.A., Xie Y., Mohr F.C., Margolin S.B., Giri S.N. (2002). Effect of pirfenidone against vanadate-induced kidney fibrosis in rats. Biochem. Pharmacol..

[124] Giri S.N., Al-Bayati M.A., Du X., Schelegle E., Mohr F.C., Margolin S.B. (2004). Amelioration of doxorubicin-induced cardiac and renal toxicity by pirfenidone in rats. Cancer Chemother. Pharmacol..

[125] Miric G., Dallemagne C., Endre Z., Margolin S., Taylor S.M., Brown L. (2001). Reversal of cardiac and renal fibrosis by pirfenidone and spironolactone in streptozotocin-diabetic rats. Br. J. Pharmacol..

[126] Shimizu T., Kuroda T., Hata S., Fukagawa M., Margolin S.B., Kurokawa K. (1998). Pirfenidone improves renal function and fibrosis in the post-obstructed kidney. Kidney Int..

[127] Salhiyyah K., Forster R., Senanayake E., Abdel-Hadi M., Booth A., Michaels J.A. (2015). Pentoxifylline for intermittent claudication. Cochrane Database Syst. Rev..

[128] Subramaniyan V., Chakravarthi S., Jegasothy R., Seng W.Y., Fuloria N.K., Fuloria S., Hazarika I., Das A. (2021). Alcohol-associated liver disease: A review on its pathophysiology, diagnosis and drug therapy. Toxicol. Rep..

[129] Strutz F., Heeg M., Kochsiek T., Siemers G., Zeisberg M., Muller G.A. (2000). Effects of pentoxifylline, pentifylline and gamma-interferon on proliferation, differentiation, and matrix synthesis of human renal fibroblasts. Nephrol. Dial. Transplant..

[130] Lin S.L., Chen Y.M., Chien C.T., Chiang W.C., Tsai C.C., Tsai T.J. (2002). Pentoxifylline attenuated the renal disease progression in rats with remnant kidney. J. Am. Soc. Nephrol..

[131] Zhang X., Meng F., Song J., Zhang L., Wang J., Li D., Li L., Dong P., Yang B., Chen Y. (2016). Pentoxifylline ameliorates cardiac fibrosis, pathological hypertrophy, and cardiac dysfunction in angiotensin ii-induced hypertensive rats. J. Cardiovasc. Pharmacol..

[132] Li H., Hua J., Guo C.X., Wang W.X., Wang B.J., Yang D.L., Wei P., Lu Y.P. (2016). Pentoxifylline inhibits liver fibrosis via hedgehog signaling pathway. J. Huazhong Univ. Sci. Technol. Med. Sci..

[133] Lin Y., Xu Z., Zhou B., Ma K., Jiang M. (2022). Pentoxifylline inhibits pulmonary fibrosis by regulating cellular senescence in mice. Front. Pharmacol..

[134] Chen Y.M., Chiang W.C., Yang Y., Lai C.F., Wu K.D., Lin S.L. (2015). Pentoxifylline attenuates proteinuria in anti-Thy1 glomerulonephritis via downregulation of nuclear factor-kappaB and Smad2/3 signaling. Mol. Med..

[135] Maruno S., Tanaka T., Nangaku M. (2022). Exploring molecular targets in diabetic kidney disease. Kidney Res. Clin. Pract..

[136] de Morales A.M., Goicoechea M., Verde E., Carbayo J., Barbieri D., Delgado A., Verdalles U., de Jose A.P., Luno J. (2019). Pentoxifylline, progression of chronic kidney disease (CKD) and cardiovascular mortality: Long-term follow-up of a randomized clinical trial. J. Nephrol..

[137] Leung A.K.L. (2015). The whereabouts of microrna actions: Cytoplasm and beyond. Trends Cell Biol..

[138] Mohr A.M., Mott J.L. (2015). Overview of microrna biology. Semin. Liver Dis..

[139] Patel A.A., Ganepola G.A.P., Rutledge J.R., Chang D.H. (2019). The potential role of dysregulated mirnas in Alzheimer’s disease pathogenesis and progression. J. Alzheimers Dis..

[140] Boon R.A., Iekushi K., Lechner S., Seeger T., Fischer A., Heydt S., Kaluza D., Treguer K., Carmona G., Bonauer A. (2013). MicroRNA-34a regulates cardiac ageing and function. Nature.

[141] Jin Z.Q. (2021). Microrna targets and biomarker validation for diabetes-associated cardiac fibrosis. Pharmacol. Res..

[142] Lee S.A., Choi C., Yoo T.H. (2021). Extracellular vesicles in kidneys and their clinical potential in renal diseases. Kidney Res. Clin. Pract..

[143] Liu S., Wu W., Liao J., Tang F., Gao G., Peng J., Fu X., Zhan Y., Chen Z., Xu W. (2022). MicroRNA-21: A critical pathogenic factor of diabetic nephropathy. Front. Endocrinol..

[144] Zarjou A., Yang S., Abraham E., Agarwal A., Liu G. (2011). Identification of a microrna signature in renal fibrosis: Role of miR-21. Am. J. Physiol. Ren. Physiol..

[145] McClelland A.D., Herman-Edelstein M., Komers R., Jha J.C., Winbanks C.E., Hagiwara S., Gregorevic P., Kantharidis P., Cooper M.E. (2015). MiR-21 promotes renal fibrosis in diabetic nephropathy by targeting pten and Smad7. Clin. Sci..

[146] Chung A.C., Lan H.Y. (2015). MicroRNAs in renal fibrosis. Front. Physiol..

[147] Zhao S., Li W., Yu W., Rao T., Li H., Ruan Y., Yuan R., Li C., Ning J., Li S. (2021). Exosomal miR-21 from tubular cells contributes to renal fibrosis by activating fibroblasts via targeting pten in obstructed kidneys. Theranostics.

[148] Chau B.N., Xin C., Hartner J., Ren S., Castano A.P., Linn G., Li J., Tran P.T., Kaimal V., Huang X. (2012). MicroRNA-21 promotes fibrosis of the kidney by silencing metabolic pathways. Sci. Transl. Med..

[149] Zhong X., Chung A.C., Chen H.Y., Meng X.M., Lan H.Y. (2011). Smad3-mediated upregulation of miR-21 promotes renal fibrosis. J. Am. Soc. Nephrol..

[150] Zhong X., Chung A.C., Chen H.Y., Dong Y., Meng X.M., Li R., Yang W., Hou F.F., Lan H.Y. (2013). miR-21 is a key therapeutic target for renal injury in a mouse model of type 2 diabetes. Diabetologia.

[151] Qin W., Chung A.C., Huang X.R., Meng X.M., Hui D.S., Yu C.M., Sung J.J., Lan H.Y. (2011). TGF-beta/Smad3 signaling promotes renal fibrosis by inhibiting miR-29. J. Am. Soc. Nephrol..

[152] Wang B., Komers R., Carew R., Winbanks C.E., Xu B., Herman-Edelstein M., Koh P., Thomas M., Jandeleit-Dahm K., Gregorevic P. (2012). Suppression of microRNA-29 expression by TGF-beta1 promotes collagen expression and renal fibrosis. J. Am. Soc. Nephrol..

